# Effects of Antitumor Necrosis Factor Therapy on Osteoprotegerin, Neopterin, and sRANKL Concentrations in Patients with Rheumatoid Arthritis

**DOI:** 10.1155/2015/276969

**Published:** 2015-10-21

**Authors:** Katharina Kurz, Manfred Herold, Elisabeth Russe, Werner Klotz, Guenter Weiss, Dietmar Fuchs

**Affiliations:** ^1^Department of Internal Medicine VI, Innsbruck Medical University, 6020 Innsbruck, Austria; ^2^Division of Biological Chemistry, Biocenter, Innsbruck Medical University, 6020 Innsbruck, Austria

## Abstract

*Background*. Rheumatoid arthritis is a systemic autoimmune disease characterized by joint erosions, progressive focal bone loss, and chronic inflammation.* Methods*. 20 female patients with moderate-to-severe rheumatoid arthritis were treated with anti-TNF-antibody adalimumab in addition to concomitant antirheumatic therapies. Patients were assessed for overall disease activity using the DAS28 score, and neopterin, erythrocyte sedimentation rate (ESR), and C-reactive protein (CRP) concentrations as well as osteoprotegerin (OPG) and soluble receptor activator of NF-*κ*B ligand (sRANKL) concentrations were determined before therapy and at week 12. Neopterin as well as OPG and sRANKL were determined by commercial ELISAs.* Results*. Before anti-TNF therapy patients presented with high disease activity and elevated concentrations of circulating inflammatory markers. OPG concentrations correlated with neopterin (*rs* = 0.494, *p* = 0.027), but not with DAS28. OPG concentrations and disease activity scores declined during anti-TNF-treatment (both *p* < 0.02). Patients who achieved remission (*n* = 7) or showed a good response according to EULAR criteria (*n* = 13) presented with initially higher baseline OPG levels, which subsequently decreased significantly during treatment (*p* = 0.018 for remission, *p* = 0.011 for good response).* Conclusions*. Adalimumab therapy was effective in modifying disease activity and reducing proinflammatory and bone remodelling cascades.

## 1. Introduction

Rheumatoid arthritis (RA) is a chronic disease characterized by systemic inflammation and progressive periarticular bone loss leading to joint-related disability. Activated macrophages, T-and B-lymphocytes, dendritic cells, and other immunocompetent cells are present in the synovia of patients with RA [1], and these cells are involved in the destruction of bone and cartilage as they produce proinflammatory cytokines like tumor necrosis factor-*α* (TNF*α*), interleukin-1 (IL-1), interleukin-6 (IL-6), and also interferon-*γ* (IFN-*γ*) [2–4]. Elevated concentrations of proinflammatory cytokines have been detected in the joints [2–5] and in the peripheral blood of patients [6–8]. These cytokines do not only enhance inflammatory cascades in RA, but also target and affect bone metabolism. This is achieved by their influence on the expression of key regulatory proteins such as osteoprotegerin (OPG), receptor activator of NF-*κ*B ligand (RANKL), and soluble RANKL (sRANKL) [3, 9]. Binding of RANK to sRANKL, which is induced by activated T-cells [10], induces the differentiation of osteoclast precursors to mature osteoclasts, and this process is strongly enhanced by the potent osteoclastogenic cytokine TNF [11]. OPG on the other hand is produced by a variety of cells and tissues, including osteoblasts and stromal cells. OPG is a natural decoy receptor for both soluble and cell-bound RANKL and efficiently inhibits osteoclast differentiation [12]. The interaction between OPG, RANKL, and RANK is very complex and disbalance in this very critical interplay is supposed to be responsible for skeletal lesions in patients with RA [13–15].

OPG and sRANKL can be detected not only in the synovial fluid, but also in the sera of RA patients. Higher serum levels of these two biomarkers of increased bone turnover were demonstrated in the sera of patients with RA in comparison to healthy individuals [9]. Another study found higher serum OPG levels in RA patients compared to patients with osteoarthritis [16]. Serum OPG levels were also associated with the radiological confirmed bone destruction in patients with RA [17].

Anti-TNF therapy with specific TNF blocking antibodies such as infliximab, adalimumab, or golimumab is a well-established treatment for RA [18, 19], as these proteins interfere with inflammatory cascades and thereby prevent or slow down joint destruction very efficiently [20]. Furthermore, anti-TNF therapy also appears to influence bone metabolism in a positive way, as bone resorption seems to be slowed down [21–23]. An earlier study showed that sRANKL concentrations were predictive of the therapeutic response to anti-TNF therapy in RA patients, while OPG blood levels were not [24].

In our study, we investigated the relationship between inflammatory markers and bone resorption markers in RA patients and how anti-TNF therapy influences inflammation and bone turnover.

## 2. Patients and Methods

### 2.1. Patients

Twenty female patients (median age 58.5 years) with long-standing rheumatoid arthritis according to the classification criteria of the American College of Rheumatology 1987 were included. All patients presented with signs of moderate-to-severe active rheumatoid arthritis and were treated with standard treatment (mostly combination therapy; methylprednisolone, *n* = 16; Pred: prednisolone, *n* = 1; MTX: methotrexate, *n* = 11; leflunomide, *n* = 9; azathioprine, *n* = 3; cyclosporine A, *n* = 3; hydroxychloroquine, *n* = 3; SSZ: sulfasalazine, *n* = 1; NSAIDs: nonsteroidal anti-inflammatory drugs, *n* = 10 [regular], *n* = 5 [on demand]; bisphosphonates, *n* = 5; and calcium and vitamin D supplementation, *n* = 9).

Patients gave informed consent that parts of their blood specimens collected during routine venipuncture were used for additional analyses within this study. When anti-TNF therapy was indicated patients received the monoclonal TNF-antibody adalimumab (40 mg subcutaneously every other week) in addition to their other disease-modifying antirheumatic drugs.

Patients were reexamined at 12 weeks; their clinical course was monitored by disease activity score on 28 joints (DAS28). Apart from standard blood examinations an additional serum sample was obtained prior to commencing adalimumab therapy (baseline) and after 12 weeks of therapy. The response to treatment was classified as remission, if DAS28 values were <2.6 after 12 weeks of adalimumab.

### 2.2. Measurements

Serum samples of patients were frozen at –20°C until analysis. Neopterin concentrations were determined by ELISA (BRAHMS Diagnostics, Hennigsdorf, Germany). Osteoprotegerin and sRANKL concentrations were measured by commercially available ELISAs (both from Biomedica, Vienna, Austria). The ratio of sRANKL/osteoprotegerin (sRANKL/OPG) was calculated to estimate bone turnover.

### 2.3. Statistical Analysis

Nonparametric tests were used for comparisons between subgroups of patients (Mann-Whitney test) and to assess therapy effects (Wilcoxon test). Spearman rank correlation analysis was employed to assess correlations; *p* values < 0.05 were considered to indicate statistical significance. Univariate binary logistic regression analysis was performed to predict treatment response.

## 3. Results

Most patients suffered from tender and swollen joints and accordingly had a moderate-to-high disease activity with a median disease activity score (DAS28) of 5.7 before adalimumab therapy. Median concentrations as well as interquartile ranges of laboratory parameters and bone resorption markers of patients before and after treatment are shown in Table 1.

Before therapy, higher ESR concentrations were seen in patients with higher DAS28 scores and more tender or swollen joints (*rs* = 0.540, *p* = 0.014 for ESR); CRP concentrations tended to be associated with DAS28 score (*rs* = 0.430, *p* = 0.059 for CRP). Neopterin levels and rheumatoid factor (RF) concentrations were not associated with disease activity. ESR levels were correlated with CRP (*rs* = 0.625, *p* = 0.003) and neopterin concentrations (*rs* = 0.449, *p* = 0.047), while no correlations were found between these inflammatory markers and RF levels.

OPG levels were correlated with neopterin concentrations (*rs* = 0.494, *p* = 0.015), but not with CRP or ESR, before therapy. There were no differences regarding bone resorption markers between patients who were under treatment with corticosteroids (*n* = 17) or between patients with bisphosphonate therapy (*n* = 5) or calcium and vitamin D supplementation (*n* = 9) in comparison to patients who did not receive that medication. Age was not correlated with bone markers; however, in six patients younger than 50 years we observed significantly higher OPG levels compared to RA patients >50 years (*p* = 0.012).

**Figure 1:**
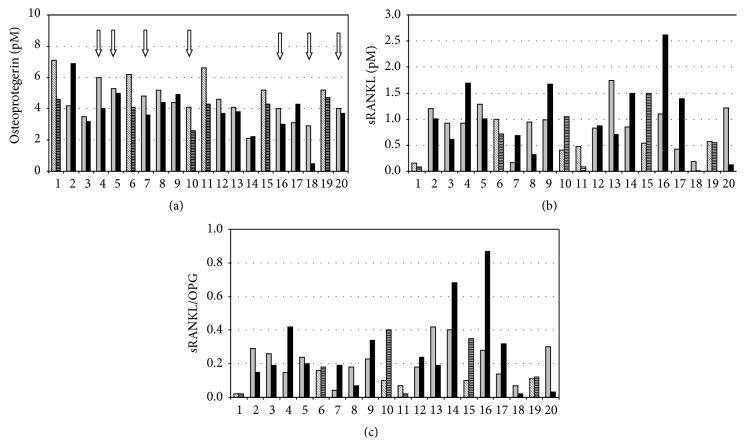
Concentrations of osteoprotegerin decreased significantly under adalimumab therapy (a), while sRANKL levels (b) and the sRANKL/OPG ratio (c) did not change. Grey columns: baseline concentrations and black columns: concentrations after 12 weeks of adalimumab treatment. In six patients <50 years (indicated by striped columns) OPG levels at baseline were significantly higher than in the other patients at baseline and decreased significantly under adalimumab treatment. sRANKL concentrations at baseline tended to be higher in older patients, and the sRANKL/OPG ratio was significantly higher in older patients. Patients who achieved remission are indicated by white arrows.

sRANKL concentrations tended to be higher in older patients (*p* = 0.069; see also Figure 1), while the sRANKL/OPG ratio was significantly higher in patients >50 years (*p* = 0.012).

Treatment with anti-TNF-antibodies was effective in reducing disease activity. After 12 weeks of treatment the number of swollen and tender joints was significantly lower (see also Table 1), and also DAS28 scores were significantly reduced, respectively (all *p* < 0.001). OPG concentrations declined significantly (*p* = 0.015 for OPG) and CRP tended to decrease (*p* = 0.058 for CRP), while neither ESR nor neopterin changed significantly (Table 1). sRANKL levels as well as sRANKL/OPG ratios were not altered by TNF-treatment.

OPG levels decreased significantly in patients younger than 50 years (*p* = 0.028) and in patients who achieved remission (*n* = 6, baseline median OPG: 4.4 pM; OPG after 12 weeks of anti-TNF-treatment 3.85 pM, *p* = 0.018; see Figure 1); ESR levels tended to decline in patients with remission (baseline median ESR: 22 mm/h; follow-up: 14 mm/h; *p* = 0.051). Neither baseline sRANKL nor sRANKL/OPG ratios differed between patients with consecutive remission and those not achieving remission or a good EULAR response.

**Figure 2:**
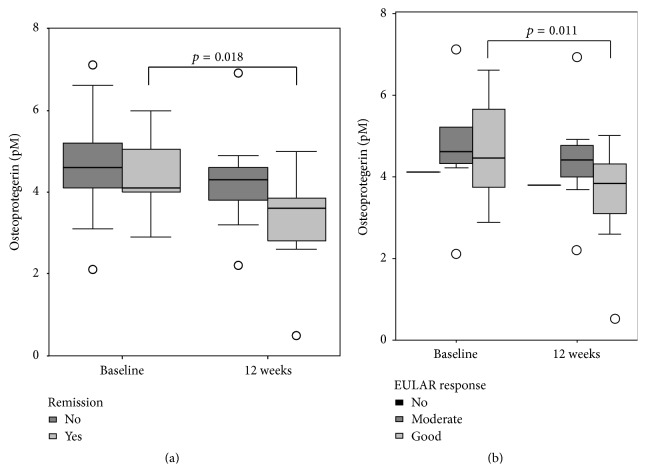
Osteoprotegerin concentrations of patients with rheumatoid arthritis according to their adalimumab treatment response: (a) compares OPG levels of patients with consecutive remission (*n* = 7, light grey boxes) to OPG concentrations of patients who did not achieve remission (*n* = 13, dark grey boxes); (b) shows that OPG concentrations decrease significantly in patients with a good EULAR response after 12 weeks of adalimumab therapy (*n* = 13, light grey boxes).

In contrast, OPG levels and CRP concentrations decreased significantly in patients with a good EULAR response (*n* = 12, *p* = 0.011 for OPG, *p* = 0.028 for CRP), while they did not change in patients with no (*n* = 1) or only a modest EULAR response (*n* = 7); see also Figure 2. None of the investigated baseline parameters differed significantly between “responders” and “nonresponders” or was predictive for consecutive remission.

**Figure 3:**
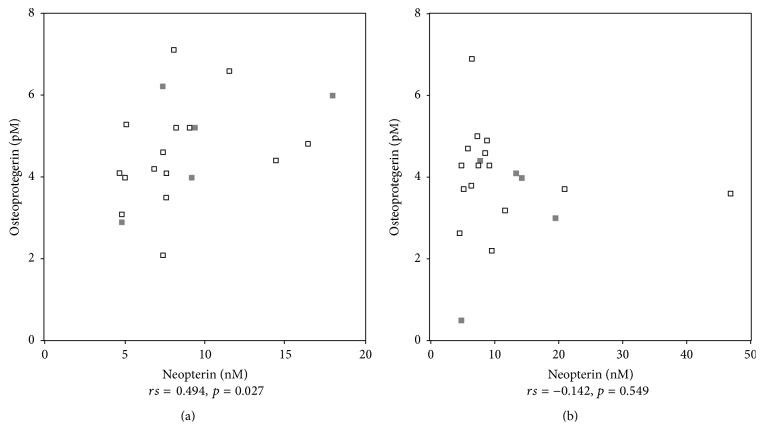
Osteoprotegerin and neopterin concentrations were correlated significantly before therapy with adalimumab (a), but not afterwards (b). Patients with additional bisphosphonate therapy are indicated by light grey filled squares.

The association between OPG and neopterin levels was not detectable anymore after 12 weeks of adalimumab therapy (see also Figure 3).

## 4. Discussion

In our study, adalimumab treatment did not only lower the number of tender and swollen joints, thus changing the severity of the symptoms, but was also effective in decreasing inflammation as reflected by significant reduction of CRP levels along with an influence on bone metabolism as indicated by decreased OPG levels. Herein, we report for the first time a correlation between baseline OPG levels and neopterin concentrations which may have practical clinical implications for easy monitoring of bone metabolism/destruction in patients with RA. Determination of neopterin is well established to estimate Th1 type immune responses in vivo [25]. Neopterin is produced by human monocyte-derived macrophages and dendritic cells (DC) upon stimulation with the proinflammatory cytokine IFN-*γ* [25]. Elevated neopterin concentrations have been described in patients with RA [26, 27]. However, recent studies are controversial regarding the role of neopterin as an activity marker of RA. Some studies could not find correlations between disease activity and neopterin levels [28, 29], while others proposed neopterin as good marker for disease activity in early RA [30] in treated RA patients [31] or showed correlations with the stage of disease [32]. CRP and ESR are measured routinely in many patients attending hospital almost worldwide whereas neopterin determination is not available everywhere. An association between CRP and ESR levels as well as neopterin concentrations has been shown already earlier in patients with other diseases such as cardiovascular disease [33] and malignancies [34, 35] as well as in patients with RA [29].

Increased serum OPG levels were correlated in earlier studies with other inflammatory markers like CRP, ESR, or rheumatoid factor [16]. However, to our knowledge this is the first time that an association between neopterin concentrations and OPG has been found. The correlation observed between serum OPG and neopterin concentrations in our study might indicate a stimulatory effect of IFN-*γ* on OPG formation or just reflect the recently proposed “antiosteoclastogenic” properties of IFN-*γ* [40]. Already in 1986, a Japanese group reported that IFN-*γ* inhibits bone resorption in part by inhibiting osteoclast formation [36]. Takayanagi and coworkers could show that IFN-*γ* strongly suppresses osteoclastogenesis by rapid degradation of the RANK adapter protein, TRAF6 (tumor necrosis factor receptor-associated factor 6), which results in strong inhibition of the RANKL-induced activation of the transcription factor NF-kappaB and c-Jun N-terminal kinases (JNK) [14]. TNF and IL-1 were shown to induce OPG formation in peripheral blood mononuclear cells and in fibroblast synovial cells earlier [9, 37], whereas IFN-*γ* inhibited both RANKL and OPG formation by fibroblast cells [37]. Furthermore TNF was also shown to induce interleukin-6 and OPG formation by synoviocytes in vitro [38], and interestingly, this effect could be increased further with patient plasma dilutions and inhibited by infliximab. The same study also could demonstrate that levels of circulating TNF bioactivity predicted clinical response to TNF inhibition [38], and similarly, also OPG formation was inhibited more strongly by infliximab in patients with a good clinical response.

IFN-*γ* and TNF can exert synergistic effects [39] which is not the case with respect to their effects on sRANKL (see also review by Zupan and coworkers [40], Kohara et al. 2011 [41]) or OPG expression [37]. This might also explain why anti-TNF-antibodies were very effective in improving patients' symptoms, while no relevant alteration of IFN-*γ* mediated tryptophan degradation was observed [29], and neopterin concentrations did not differ before and under adalimumab treatment in our patients. Anti-TNF-treatment inhibits bone resorption [42]. Thus, also elevated OPG levels in RA patients compared to healthy controls drop in response to treatment with biologicals [9].

In line with earlier studies [9, 24] we could find decreasing OPG levels under adalimumab treatment. However, sRANKL concentrations did not normalize in our patients, while they dropped significantly in 43 RA patients in the study by Ziolkowska and coworkers and decreased slightly in 75 RA patients treated with biologicals [24]. Like Ziolkowska we could not find a correlation of OPG and sRANKL levels or the OPG/sRANKL ratio with disease activity (DAS28), whereas González-Alvaro et al., 2007, reported a mild, but significant association between RANKL and DAS28 score [24]. The same group also reported a correlation of OPG levels with patients' age [24], which could not be demonstrated in our population, nor in another study [9]. Interestingly, in our study we could also confirm higher OPG levels in six patients younger than 50 years compared to older patients as demonstrated earlier by Ziolkowska et al. 2002 [9]. Ziolkowska and coworkers suggested that in older RA patients OPG might reach maximum levels characteristic for their age and that additional disease-related factors would exert only minor additive effects. In fact, this explanation would fit well with our finding that OPG levels dropped significantly and rather strongly in all younger patients under adalimumab, while in the older group they only tended to decrease (see also Figure 1). Unfortunately, we could not determine OPG and sRANKL levels in healthy individuals; however, earlier studies could show that aging goes along with increasing OPG levels [43, 44].

OPG concentrations declined significantly in patients who achieved remission or a good EULAR response, while CRP levels declined in patients with consecutive good EULAR response. ESR levels and neopterin concentrations did not change. None of the investigated baseline parameters was predictive for remission or a good EULAR response in our study, while in another study lower baseline sRANKL levels and OPG/sRANKL ratio were proposed to predict remission under anti-TNF-treatment [24].

Our study is limited by the rather low number of patients investigated thus far. Still, the associations found are of interest and especially the correlation observed between OPG and neopterin levels deserves further investigation. Further longitudinal clinical studies are needed to better characterize the relationship between inflammation/immune activation and bone resorption in patients with RA and especially to establish which markers might be suited to predict treatment response or identify patients at risk for relapse. A recent review by Dénarié and coworkers [45] overviews biomarkers of bone, cartilage, or synovium turnover and concludes that although several biomarkers correlate with RA activity, they are only of limited value to predict relapse. Thus, certainly more studies are needed to evaluate the suitability of such biomarkers as well as of other inflammation markers in remission and possibly find a good prognostic biomarker for the management of rheumatoid arthritis.

## 5. Conclusions

Therapy with adalimumab does not only improve disease activity, but also seems to influence inflammatory and bone remodelling cascades in patients with long-standing RA. OPG levels might be useful to predict remission or clinical response.

## Figures and Tables

**Table 1:** Median (and interquartile ranges in brackets) number of tender and swollen joints and DAS28 score as well as the median concentrations of investigated lab parameters before and under adalimumab therapy (*n* = 20).

	Baseline	After adalimumab therapy, week 12	*p* value
DAS28 score	5.7 (5.4–6.3)	3.1 (2.4–3.7)	<0.001
Tender joints	12 (7–17)	0 (0–2)	<0.001
Swollen joints	9 (6–11)	1 (0–4)	<0.001
Neopterin (nM)	7.6 (5.5–9.4)	8.2 (6.0–13.0)	n.s.
C-reactive protein (mg/L)	13.1 (6.2–31.1)	5.8 (0.9–16.3)	0.058
ESR (mm/h)	31 (17–51)	23 (11–41)	n.s.
Osteoprotegerin (pM)	4.5 (4.0–5.3)	4.1 (3.3–4.6)	0.015
sRANKL (pM)	0.89 (0.44–1.08)	0.80 (0.38–1.47)	n.s.
sRANKL/OPG	0.17 (0.1–0.28)	0.19 (0.8–0.35)	n.s.
